# Pathophysiological Integration of Metabolic Reprogramming in Breast Cancer

**DOI:** 10.3390/cancers14020322

**Published:** 2022-01-10

**Authors:** Roberto Corchado-Cobos, Natalia García-Sancha, Marina Mendiburu-Eliçabe, Aurora Gómez-Vecino, Alejandro Jiménez-Navas, Manuel Jesús Pérez-Baena, Marina Holgado-Madruga, Jian-Hua Mao, Javier Cañueto, Sonia Castillo-Lluva, Jesús Pérez-Losada

**Affiliations:** 1 Instituto de Biología Molecular y Celular del Cáncer (IBMCC-CIC), Universidad de Salamanca/CSIC, 37007 Salamanca, Spain; rober.corchado@usal.es (R.C.-C.); nataliagarciasancha@usal.es (N.G.-S.); marinamendiburu@usal.es (M.M.-E.); a.gomezvecino@usal.es (A.G.-V.); ajimeneznavas@usal.es (A.J.-N.); mjperezbaena@usal.es (M.J.P.-B.); jcanueto@usal.es (J.C.); 2 Instituto de Investigación Biosanitaria de Salamanca (IBSAL), 37007 Salamanca, Spain; mholgado@usal.es; 3 Departamento de Fisiología y Farmacología, Universidad de Salamanca, 37007 Salamanca, Spain; 4 Instituto de Neurociencias de Castilla y León (INCyL), Universidad de Salamanca, 37007 Salamanca, Spain; 5 Biological Systems and Engineering Division, Lawrence Berkeley National Laboratory, Berkeley, CA 94720, USA; jhmao@lbl.gov; 6 Berkeley Biomedical Data Science Center, Lawrence Berkeley National Laboratory, Berkeley, CA 94720, USA; 7 Departamento de Dermatología, Hospital Universitario de Salamanca, Paseo de San Vicente 58-182, 37007 Salamanca, Spain; 8 Complejo Asistencial Universitario de Salamanca, 37007 Salamanca, Spain; 9 Departamento de Bioquímica y Biología Molecular, Facultad de Ciencias Químicas, Universidad Complutense, 28040 Madrid, Spain; 10 Instituto de Investigaciones Sanitarias San Carlos (IdISSC), 28040 Madrid, Spain

**Keywords:** metabolism, interstitium, glucose, lactate, hypoxia, cancer-associated fibroblasts, macrophages

## Abstract

**Simple Summary:**

Tumors exhibit metabolic changes that differentiate them from the normal tissues from which they derive. These metabolic changes favor tumor growth, are primarily induced by cancer cells, and produce metabolic and functional changes in the surrounding stromal cells. There is a close functional connection between the metabolic changes in tumor cells and those that appear in the surrounding stroma. A better understanding of intratumoral metabolic interactions may help identify new vulnerabilities that will facilitate new, more individualized treatment strategies against cancer. We review the metabolic changes described in tumor and stromal cells and their functional changes and then consider, in depth, the metabolic interactions between the cells of the two compartments. Although these changes are generic, we illustrate them mainly with reference to examples in breast cancer.

**Abstract:**

Metabolic changes that facilitate tumor growth are one of the hallmarks of cancer. The triggers of these metabolic changes are located in the tumor parenchymal cells, where oncogenic mutations induce an imperative need to proliferate and cause tumor initiation and progression. Cancer cells undergo significant metabolic reorganization during disease progression that is tailored to their energy demands and fluctuating environmental conditions. Oxidative stress plays an essential role as a trigger under such conditions. These metabolic changes are the consequence of the interaction between tumor cells and stromal myofibroblasts. The metabolic changes in tumor cells include protein anabolism and the synthesis of cell membranes and nucleic acids, which all facilitate cell proliferation. They are linked to catabolism and autophagy in stromal myofibroblasts, causing the release of nutrients for the cells of the tumor parenchyma. Metabolic changes lead to an interstitium deficient in nutrients, such as glucose and amino acids, and acidification by lactic acid. Together with hypoxia, they produce functional changes in other cells of the tumor stroma, such as many immune subpopulations and endothelial cells, which lead to tumor growth. Thus, immune cells favor tissue growth through changes in immunosuppression. This review considers some of the metabolic changes described in breast cancer.

## 1. Metabolic Changes in Tumor Cells

Proliferating tumor cells require large quantities of energy. High rates of proliferation, originating from mutations in genes that regulate cell growth, is one of the characteristics of tumor tissue. Tumor cells require more energy and molecules to build cell structures by an anabolic process to achieve this high degree of proliferation. Thus, proliferating tumor cells undergo metabolic changes to increase the acquisition of energy and to activate anabolic reactions. However, these metabolic changes initially consist of the activation of glycolysis and the inhibition of oxidative phosphorylation (OXPHOS), together known as the Warburg effect [1]. A two-compartment model, otherwise known as the inverted Warburg (or coupling) model, was subsequently proposed, in which tumor parenchyma proliferation is maintained by the glycolytic activity of the stroma. This model explains the existence of tumors with high levels of mitochondrial respiration and low rates of glycolysis [2,3,4]. Indeed, cancer cells can maintain high levels of tricarboxylic acid (TCA) cycle activity in many tumors, for whom inhibition has been proposed as a treatment strategy [5]. However, the scenario may be more complex, such that the two energy-gathering systems can coexist in tumor cells. Indeed, cancer cells can swap from one energy-gathering system to another and switch from glycolysis to OXPHOS even under conditions of lactic acidosis [6]. In the context of breast cancer, this mixed metabolism is more frequent in triple-negative breast tumors than in luminal tumors in which glycolysis would predominate [7].

In any case, the surrounding tumor stroma is reprogrammed to support the proliferation of the tumor parenchyma. These changes in stromal function derive from the metabolic activity of the tumor cells and translate into two main events: first, an increase in the consumption of nutrients that they extract from the environment and, second, an increase in the release of metabolites into the microenvironment [8,9].

This review describes some of the changes that occur in tumor cells, then discusses those observed in some stromal subpopulations. Finally, we examine how these changes are integrated into the crosstalk between the two main tumor compartments: cancer cells and the stroma. We will refer to cancer in general, although most of the examples mentioned are in breast cancer.

### 1.1. Tumors Can Show Increased Glycolysis, Decreased Krebs Cycle Activity, and Increased Acidification of the Interstitium Due to Lactate Release

Tumor cells voraciously take up glucose, their primary carbon source, from the interstitium. Its continuous uptake by the cells makes it scarce in the microenvironment [8,10]. Glucose processing through the aerobic glycolysis pathway involves the propensity for proliferating cells, including cancer cells, to take up glucose and secrete carbon as lactate, even when oxygen is present. Thus, glucose processing through aerobic glycolysis provokes an increase in lactate production because pyruvate is preferentially transformed into lactate rather than passing to acetyl-CoA and entering the Krebs tricarboxylic acid (TCA) cycle. The decrease in the activity of the TCA cycle leads to a reduction in mitochondrial oxidation; this is known as the Warburg effect, which was first described in 1927 [1]. However, as indicated above, this classical interpretation of the metabolic changes in cancer is not always accurate; some modifications and compartmentalization have been introduced more recently [2], as discussed below. The glycolysis is present in the stromal cells, and the TCA and OXPHOS activities can be increased in tumors cells. Indeed, ATP generation based on glycolysis or OXPHOS depends on tumor type, grade, and the stage of tumor progression. Both energy-gathering systems can be present in tumor cells, and even cancer cells can move from one energy-gathering system to another [11].

As a consequence of the high level of aerobic glycolysis in the stroma and, at times, in tumor cells, there is a copious release and accumulation of lactate in the tumor interstitium. Excess lactic acid is released by tumor and stromal cells into the extracellular environment through specific transporters such as monocarboxylate transporter (MCT) systems. Lactate accumulation leads to the acidification of the microenvironment [12]. The ineffective clearance of lactate also contributes to this due to the inadequate perfusion of the tumor tissue. Thus, in the tumor interstitium, a concentration of up to 40 mM of lactate is detected. A high lactate concentration in the interstitium has been associated with an increased risk of metastasis and death from cancer [13]. However, although less effective than glucose, lactate is also an energy source for physiological and tumor processes [14,15].

Regarding breast cancer: As expected, high levels of glycolytic activity have also been demonstrated in breast cancer tissue and cell lines [16,17,18,19]. High glycolysis levels have been associated with increased cell proliferation [20]. Increased glucose uptake and overexpression of glycolytic pathway enzymes contribute to glycolytic activity. An increase in glucose uptake through the GLUT1 transporter, which is overexpressed in triple-negative breast cancer of basal phenotype [21], is correlated with poor prognosis [22]. GLUT1 and GLUT3 glucose transporters are more overexpressed in higher-grade breast cancer (grades 2 and 3) than in grade 1 [23]. Increased glycolytic activity is also explained by the greater activity of some of the enzymes in the pathway, such as hexokinase 2, which is overexpressed in breast cancer [24,25] and whose inhibition in transgenic mice that develop breast cancer after overexpression of *ErbB2/Neu* delays tumor development [24]. Raised levels of phosphofructokinase have also been detected in breast cancer [26,27], as have those of pyruvate kinase M2, whose overexpression is associated with reduced survival and increased risk of metastasis in breast cancer [28,29].

The low level of activity of TCA and the mechanism by which pyruvate does not enter the TCA in the tumors that occur are not fully understood. In breast cancer, it could be explained by a low level of expression of the PDHX component of the pyruvate dehydrogenase (PDH), which is an enzyme that controls the flow of metabolites from glycolysis to TCA. This low level of PDHX expression is associated with poor survival in breast cancer [30]. There are also differences in the expression levels of some of the enzymes that participate in TCA between breast cancer subtypes. For example, the expression levels of the A subunit of succinate dehydrogenase are higher in HER2-positive tumors than in luminal A tumors [31,32].

The pentose phosphate pathway (PPP) is another glucose oxidation pathway, in addition to glycolysis and the TCA. It is enhanced in tumors, leading to the generation of lipid and nucleotide precursors (ribose phosphate) that are subsequently used to synthesize macromolecules and of NADPH, which is necessary to protect tumor cells from the large amount of radical oxidative species (ROS) they produce [33].

Given the importance of PPP in tumor proliferation and survival of the action of ROS, it is not surprising that high levels of some PPP enzymes, such as glucose 6-phosphate dehydrogenase and transketolase, are associated with poor evolution in breast cancer [34]. Specifically, some PPP enzymes are mainly expressed in HER2+ tumors, suggesting that the activation of this pathway is essential in this intrinsic subtype of breast cancer [7].

### 1.2. Cancer Cells Use More Glutamine and Other Amino Acids

Amino acids are the second carbon source for proliferating tumor cells, providing energy and making up most of the biomass of proliferating tumor cells [35]. Therefore, cancer cells massively capture amino acids from the interstitium. This uptake is indirectly mediated by proliferating activators such as Myc [36]. The most important of these amino acids is glutamine (Gln), which has several roles in tumor cells: (i) it is an intermediate metabolite for synthesizing nucleotides and non-essential amino acids (NEAAs); (ii) it allows the uptake of other essential amino acids (EAAs) by transporters in an antiport manner, whereby Gln is expelled from the cell in exchange for capturing another amino acid; (iii) it has a role in regenerating intermediate metabolites of TCA in an anaplerotic reaction [37]; (iv) it is important in the synthesis of glutathione, which is essential to avoid excess intracellular oxidation and to maintain the reduction–oxidation (redox) state [38,39,40].

The significant proliferation of tumor cells leads to a strong uptake of amino acids from the interstitium in general, not just glutamine [41,42,43], giving rise to a shortage of NEAAs in the interstitium, such as glutamine, serine, and cysteine [8]. Serine is necessary for synthesizing nucleotides and cysteine, like glutamine, to synthesize glutathione [44]. For this reason, tumor cells have developed different strategies to ensure the supply of amino acids by, for example, overexpressing different transporters or by the uptake of amino acids by micropinocytosis of extracellular proteins [8,45]. Indeed, protein macromolecules obtained from the degradation of the extracellular matrix (ECM) are taken up by micropinocytosis. This mechanism has been observed in tumors with mutations in *Ras* or *c-Src* [46]. Lysosomes digest the contents of these macrovesicles by releasing independent amino acids to support cell survival and proliferation under conditions of amino acid deficiency [47]. In a third strategy, tumor cells catabolize extracellular matrix proteins to obtain the amino acids they require when the latter become scarce in the interstitium [48,49]. Finally, autophagy is activated in myofibroblastic cancer-associated fibroblasts (CAFs), whose protein catabolism amino acids are released into the extracellular space and taken up by tumor cells [50,51]. As we will see later, autophagy in CAFs is essential in the metabolic exchanges between tumor cells and CAFs.

Regarding breast cancer: Glutamine is also essential in breast cancer. Glutaminase, an enzyme that converts glutamine into glutamic acid, is overexpressed in breast cancer, especially in triple-negative breast cancer (TNBC) tumors compared with HER2 and luminal subtypes [52]. Exogenous glutamine is essential for the survival of TNBC cells [53]. Luminal tumors are less dependent on exogenous glutamine, not so much because of their lower proliferation but rather because the luminal cells themselves can synthesize the amino acid by expressing a glutamine-synthetase enzyme [54].

Serine, a non-essential amino acid that can be synthesized in the organism, is another important amino acid in breast cancer. 3-phospho-glycerate-dehydrogenase (PHGDH) is the first enzyme to be involved in serine synthesis. It is overexpressed in breast cancer [55] and breast cancer cell lines [56] and, fundamentally, in the subtypes of breast cancer that proliferate more significantly, such as ER-negative [57]. The overexpression of PHGDH and the synthesis of serine are associated with tumor growth for several reasons. Serine (i) feeds the pathways of protein synthesis; (ii) influences the contribution of metabolites to TCA since it is an anaplerotic metabolite; (iii) favors the synthesis of the oncometabolite 2-hydroxy-glutarate (2HG); and (iv) fuels the one-carbon metabolism (which includes nucleic acid synthesis via folate, antioxidant defense, and methylation reactions) [58].

### 1.3. Tumor Cells Capture Large Amounts of Fatty Acids and Synthesize Complex Lipids to Construct the Cell Membrane

Proliferating tumor cells require more lipids fundamentally to build cell membranes. Increases in exogenous lipids and cholesterol uptake and their internal synthesis [59] have been described in breast cancer [60]. Focusing on breast cancer, concerning increased fatty acid (FA) synthesis, the limiting enzyme is fatty acid synthase (FAS), encoded by the *FASN* gene, which is known to have oncogenic functions [61]. Increased synthesis of fatty acids is achieved by augmented FAS expression by the *FASN* gene, which is notably overexpressed in breast cancer. Moreover, FAS levels are associated with tumor recurrence and worse prognosis [62]. FAS expression has some specificity to breast cancer subtypes. Indeed, FAS is significantly elevated in HER2+ tumors but, paradoxically, is poorly expressed in TNBC tumors despite being an aggressive subtype. This apparent anomaly could be explained by the direct activation of FAS expression by the HER2 pathway. The HER2–*FASN* axis would favor proliferation, dissemination, and resistance to chemotherapy in these tumors [63]. Thus, the level of FAS is significantly elevated in HER2+ tumors [64,65], enhancing the function of the HER2 receptor and cell proliferation [65].

Apart from HER2, *FASN* expression is regulated by other pathways. Some are explicitly related to lipid synthesis, such as SREBP-1 (sterol regulatory-binding protein-1) that binds to the *FASN* promoter [66]. *FASN* expression can also be regulated by PI3K/AKT/mTOR and MAPK [67,68], whose inhibition reduces *FASN* expression in breast cancer cells [69]. Additionally, under hypoxic conditions, *FASN* is overexpressed by the coordinated action of SREBP-1 and AKT [70]. In addition to the need for lipids for membrane synthesis, tumor cells can change their mechanisms for acquiring energy from glycolysis to a lipid-dependent form [71].

As mentioned above, there is an increase in the uptake of lipids and molecules that ends up forming part of their structure. For example, an increase in choline uptake occurs in luminal tumors and in TNBC [72,73], where it is then converted to phosphocholine and phosphatidylcholine. Subsequently, phospholipase D (PLD) excises phosphatidylcholine into phosphatidic acid and choline. The former is known to enhance the metastatic capacity of breast cancer cells [74]. PLD is overexpressed in breast cancer cells [75], and its levels are associated with the proliferative activity of tumor cells [74].

The captured fatty acids are used rapidly in the synthesis of complex lipids, such as the ceramides and triacylglycerides that form part of the structure of phospholipids and sphingolipids in cell membranes [60]. They also participate in the synthesis of inflammation mediators, such as prostaglandins. Not all fatty acids are equally used, and the predominant destinations of some fatty acids, such as palmitic acid, vary depending on the breast cancer subtype. Thus, while in TNBC, palmitate is part of the triglycerides; it preferentially enters the oxidation of fatty acids in luminal tumors [76,77]. In any case, the massive uptake of free fatty acids by tumor cells makes their levels in the interstitium low [18].

Some enzymes involved in fatty acid synthesis are essential for carcinogenesis induced by specific oncogenes. In this sense, the acyl-coenzyme A (CoA) synthetase long-chain family member 3 (ACSL3), which converts fatty acids into fatty acyl-CoA esters, may be essential in KRAS-mediated carcinogenesis [78].

Fatty acids play an essential role in resistance to chemotherapy, acting in several ways. First, tumor cells have more and bigger lipid droplets than normal cells, and they increase the area of contact between the lipid droplets and mitochondria, which facilitates the oxidation of fatty acids. Second, the previously described increase in de novo synthesis of fatty acids by augmented expression of FASN and acetyl-CoA carboxylase (ACC) contributes to treatment resistance. Third, there is an increase in the quantity of saturated fatty acids in the plasma membrane that form part of the glycerophospholipids. These reduce the fluidity of the membrane, leading to a lower rate of endocytosis and passive diffusion of anticancer drugs, as well as low ROS production and a low risk of cell death by apoptosis and ferroptosis. Finally, the increased saturated fatty acids in the glycerophospholipids also ensure the production of a significantly larger amount of detergent-resistant membrane domains, which activate the function of the pumps involved in the ABC family that cause multiple drug resistance [79].

### 1.4. Tumor Cells Adapt to a Chronic Deficit of Nutrients and Oxygen in the Interstitium

As previously indicated, tumor cells take up a large amount of glucose and NEAAs due to their substantial proliferation requirements, producing a shortage of them in the interstitium. Derived from their metabolism, they release a large amount of lactate and ammonia into the medium, which becomes acidic. The lack of nutrients and the acidification of the environment are both enhanced by reducing the functionally adequate vascularization in the tumor, which restricts the supply of nutrients and oxygenation and is incapable of effectively draining the waste products from the interstitium [80].

Tumor cells must adapt to a chronic lack of nutrients in the interstitium, derived from their activity, for which they show high metabolic plasticity and initiate a series of alternative responses. The very limited availability of glucose means that tumor cells can use acetate to synthesize acetyl-CoA and fatty acids in the absence of lipids [81]. The scarcity of lipids means that they can also collect lysophospholipids from the microenvironment [82]. The shortage of amino acids enables cancer cells to degrade extracellular proteins and capture amino acids by micropinocytosis, thereby maintaining their contribution [8,45]. They can also use branched-chain amino acids to synthesize NEAAs and nucleotides [8].

Continuously proliferating tumor cells suffer from a relative deficiency of nutrients and a lack of oxygen due to their great requirements, which adds to the vascular deficit [83,84,85]. This triggers a series of compensatory responses to the lack of oxygen. Normal cells stop proliferating under hypoxic conditions [86], but tumor cells can proliferate under conditions in which the growth of normal tissues is paralyzed. Tumor cells grow under conditions of relative oxygen deficiency because: (i) oncogenic activation forces glucose uptake; (ii) hypoxia stabilizes HIF1α; and (iii) tumor cells compete with stromal cells for oxygen, leaving them in a hypoxic state.

However, not all tumor cells continue to proliferate. Like normal cells, some stop proliferating due to the relative lack of nutrients and enter G0. This is the case for quiescent cancer stem cells, which, in G0, are relatively insensitive to glucose deprivation [87], and preferentially use lactate as an energy source, favoring their long-term survival [88,89,90,91]. This hypothesis is supported by the observation that chemotherapy-resistant cancer stem cells oxidize lactate [92].

In the specific context of breast cancer, a symbiosis or crosstalk has been described between oxygenated and non-oxygenated areas of the tumor that compensate for the deficit of nutrients and oxygen. Thus, in tumor cells in hypoxic areas, HIF1α and the expression of the lactate transporter MCT4 (monocarboxylate transporter-4) are induced. In the absence of oxygen, they are cells with a high level of glycolytic activity that produce a large quantity of lactate and release it into the interstitium through the MCT4 transporter. Lactate is taken up by tumor cells in well-oxygenated areas, which are HIF1α-negative and express the MCT1 transporter. These oxygenated tumor cells take up the lactate and use it, even in preference to glucose. Thus, it has been proposed that cells from the well-oxygenated areas require less glucose than is available to the more inadequately oxygenated cells, thereby facilitating the growth of the well and poorly oxygenated tumor areas [93,94,95].

MCT4 transporter ablation has been shown to prevent resistance to antiangiogenic treatment of breast cancer in a mouse model [94]. In breast cancer, inhibition of the lactate transporter MCT1 reduces the formation of mammospheres [96], implying that lactate uptake favors tumor initiation. Lactate also functions as a signaling metabolite, favoring angiogenesis in breast cancer [97].

### 1.5. Mutations in Breast Cancer and Metabolic Changes

Any mutation that induces proliferation in tumor cells indirectly induces metabolic reprogramming, so the two processes become coupled. However, mutated genes that control proliferation can also directly influence metabolic changes by specific mechanisms. Thus, metabolic changes can be directly induced after the gain (oncogenes) or loss (suppressors) of gene function. For the sake of conciseness, we will solely consider some essential mutations in breast cancer.

#### 1.5.1. P53

P53 is frequently mutated in breast cancer, especially in aggressive intrinsic subtypes, such as HER2+ and TNBC [98]. P53 is a tumor suppressor involved in cell-cycle arrest, which explains why the metabolic changes induced by the wild-type form of P53 aim to prevent or reduce cell proliferation. Glycolysis inhibition is one of the antiproliferative metabolic changes induced by wild-type P53. To achieve this, P53 suppresses the expression of membrane transporters of glucose, such as GLUT1, GLUT3, and GLUT4 [99,100], and regulates a series of glycolysis enzymes to limit the activity of this pathway. These enzymes include hexokinase 2 (HK2) [100], phosphofructokinase 1 (PFK1) [101], pyruvate dehydrogenase [101], and others [102,103]. Wild-type P53 also exerts its antiproliferative action by inhibiting other pathways, such as the mTOR pathway, the PPP (by inhibiting glucose-6 phosphate dehydrogenase), and fatty acid synthesis [104,105].

The metabolic brakes on cell proliferation induced by P53 are lost when P53 function is damaged by mutations. Thus, P53 inhibition of glycolysis disappears, and there is an increase in glucose uptake after activation of the RhoA/ROCK/GLUT1 cascade [102]. In addition, P53 enhances the PPP by ceasing to inhibit glucose-6-phosphate dehydrogenase [106]. Globally, with the loss of P53 function, anabolic pathways are enhanced [107]. These include fatty acid synthesis via SREBPs [108]. Conversely, catabolic pathways, such as fatty acid oxidation [107], are repressed. P53 mutations in breast cancer are also associated with the increased expression of genes involved in the mevalonate pathway, which is essential for synthesizing cholesterol [109].

#### 1.5.2. C-MYC

c-MYC activity is associated with increased cell proliferation, tumor growth, and greater metastatic capacity, along with resistance to endocrine treatment in breast cancer, leading to a worse clinical outcome [110]. Indeed, the effects of MYC mainly occur in more aggressive intrinsic subtypes of breast cancer, such as the luminal B, HER2 +, and TNBC forms [111].

To promote cell proliferation, MYC intervenes in several metabolic pathways. Fundamentally, MYC participates in reprogramming glutamine metabolism to increase TCA activity in tumor cells. Thus, MYC favors glutamine uptake after inducing the synthesis of glutamine transporters and induces the synthesis of enzymes that participate in glutamine metabolism [112]. Besides glutamine uptake, MYC also favors the uptake of other amino acids such as serine, glycine, and tryptophan [113].

MYC usually participates in tumor metabolism in combination with other molecules and pathways, such as mTOR and HIF [114,115]. Additionally, in luminal tumors, Erα induces MYC expression, which, in turn, participates as a cofactor of Erα, favoring tumor proliferation [116]. The crosstalk described between HER2 and Erα in luminal tumors enhances glutamine metabolism through MYC [117]. In TNBC tumors, MYC enhances glycolysis by inhibiting thioredoxin-interacting protein (TXNIP), a glycolysis inhibitor [118].

#### 1.5.3. ERα

ERα is a molecule that is critical to the metabolic changes of cells in breast cancer. Two-thirds of breast tumors are ERα+ [119]. ERα acts in combination with another series of molecules and signaling pathways, such as those already discussed for P53, MYC, PI3K/AKT/mTOR, and HIF [120].

Estradiol (E2) binds to ERα and enhances glycolysis by increasing the expression of the GLUT1 transporter [121]. However, the reprogramming of metabolism by the E2/ERα varies depending on glucose availability: if it is high, glycolysis is enhanced, via AKT, and TCA activity decreases, whereas if it is low, glycolysis decreases and TCA activity is enhanced instead by increasing pyruvate–dehydrogenase (PDH) activity [122].

#### 1.5.4. HER2

HER2+ tumors have a glycolytic phenotype [123,124]. Certainly, HER2 favors the use of glucose [125] by regulating different enzymes such as lactate dehydrogenase (LDH) [126] and 6-phospho-fructo-2 kinase [125]. Additionally, HER2 inhibition through the action of a dual EGFR/HER2 inhibitor leads to a low level of cell proliferation by the depletion of hexokinase-2 [127].

The potential capacity of HER2 to translocate into the mitochondria by the action of mitochondrial HSP70, where it inhibits oxygen consumption, helps enhance glycolysis [128]. Due to this glycolytic activity, lactic acid accumulation is favored in HER2+ tumors [129].

With respect to lipid metabolism, the expression of FASN is directly activated by the HER2 pathway, leading to an increase in fatty acid synthesis (see above) [63].

#### 1.5.5. BRCA1

*BRCA1* mutations are associated with triple-negative breast cancers, which are highly aggressive, high-grade tumors that feature aneuploidies and are generally associated with poor prognosis [130,131,132].

Mutations in *BRCA1* induce the generation of hydrogen peroxide in tumor cells and in the stroma. Hydrogen peroxide stimulates the transformation of fibroblasts into CAFs and the generation of a glycolytic stroma [133]. Indeed, mutations of the *BRCA1* suppressor gene, like other oncogenes (RAS, TGFβ, NFKB), are known to induce the phenotype of metabolic symbiosis between tumor cells and tumor-associated fibroblasts [134] (see below).

#### 1.5.6. PI3K/AKT/mTOR

The PI3K/AKT/mTOR signaling pathway has protumoral effects that result in tumor progression [135]. The metabolic changes that PI3K/AKT/mTOR induce promote tumor growth. Thus, the PI3K/AKT/mTOR pathway favors glycolysis and glucose uptake and the induction of HIF1α, independently of hypoxia [136,137,138,139]. It also induces the expression of genes involved in lipogenesis through SREBP [140,141] and anabolic processes through the PPP [142].

Proliferating cells require a large amount of glucose and cytoskeleton remodeling. The PI3K pathway integrates both processes. The pathway is activated in response to the insulin receptor and other growth factors. Phosphatidyl-inositol triphosphate is generated and incorporated into the plasma membrane’s inner face, where it captures different signaling proteins. One of these proteins, AKT, is involved in the assimilation and phosphorylation of glucose [143].

In contrast, the RHO/RAC/CDC42 pathway initially involves cytoskeleton remodeling [144]. However, it has recently been shown that cytoskeleton remodeling releases aldolase A, which is involved in glycolysis. The complete activation of glycolysis by the PI3K pathway requires both AKT and RAC, allowing coordination between cytoskeletal remodeling and glycolysis and, therefore, between cell division and macromolecule synthesis [145].

PI3KCA mutations in breast cancer occur in 36% of ERα+ HER2- tumors [146]. Indeed, there is crosstalk between the two pathways, whereby ERα stimulates the PI3K/AKT/mTOR pathway, favoring migration and tumor invasion [147], and the PI3K/AKT/mTOR pathway activates the expression of ERα [148,149].

**Figure 1:**
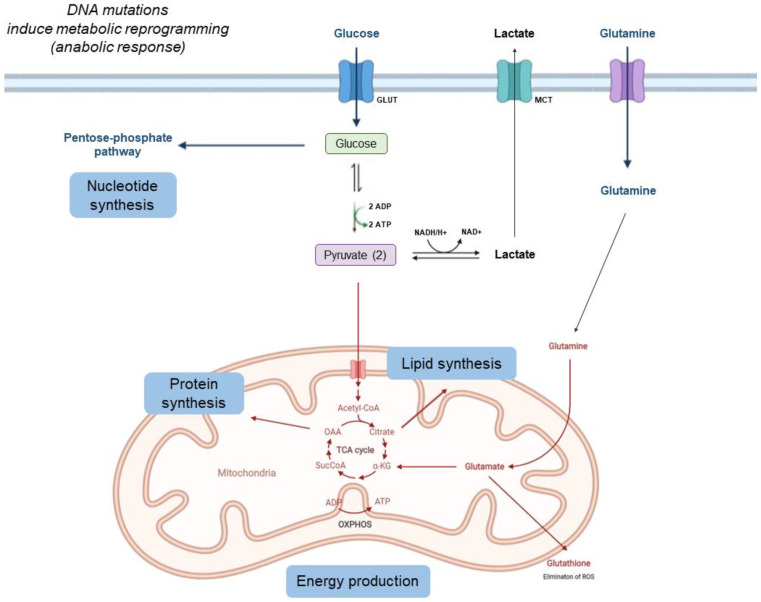
Resume of some of the metabolic changes taking place in tumor cells. During tumor development, a series of metabolic changes that favor its growth occurs. Globally, proliferating tumor cells have a great appetite for glucose and amino acids, causing a relative deficit of both in the tumor interstitium. In addition, pyruvate enters the tricarboxylic acid cycle with difficulty and is mainly transformed into lactate (Warburg effect). However, having an ATP production more based on glycolysis or OXPHOS depends on tumor type, grade, and even the stage of progression of the disease. In fact, both energy-gathering systems can coexist in tumor cells, and even cancer cells can move from one energy-gathering system to another. Created using BioRender.

Some of the main metabolic changes presented by tumor cells are shown in Figure 1.

## 2. Metabolic Changes in the Interstitium Cells

Unlike cells of the tumor parenchyma, the stromal cells of the interstitium have no great capacity for metabolic adaptation and continue to depend on the existence of nutrients in the interstitium for their survival. The scarcity of these, together with acidosis, leads to differentiation and functional changes of stromal cells that favor the growth of tumor cells. We describe below the changes that take place in some of the central stromal cell populations.

### 2.1. Metabolic Changes in Non-Leukocyte Stromal Cells

A series of non-leukocyte cell subpopulations in the tumor interstitium contributes to tumor growth, such as fibroblasts, adipocytes, and endothelial cells. In this section, we point out some of the metabolic and functional changes in these cell subpopulations in the context of cancer development.

#### 2.1.1. Metabolic Changes in Fibroblasts

Fibroblasts are the most common cells in the stroma. In the context of tumor growth, they are functionally activated and transformed into myofibroblasts, which express smooth muscle actin (SMA) alpha. These myofibroblasts, in the context of the tumor, are called cancer-associated fibroblasts (CAFs).

The low glucose concentration in the interstitium and the Warburg effect lead to less production of ATP in the CAFs. Low levels of ATP yield a higher AMP/ATP ratio, thereby activating AMPK, which has a series of effects, such as autophagy and the secretion of NEAAs into the interstitium. These NEAAs are taken up by tumor cells so that they can proliferate [50,51].

Aerobic glycolysis of CAFs produces a large quantity of lactate, which is released into the interstitium by the MCT4 transporter. When there is a glucose shortage, tumor cells also use lactate in the interstitium as an energy source, capturing it through the MCT1 transporter [150,151]. Therefore, the effect of CAFs on promoting tumor cell growth arises, at least in part, from their secretion of lactate [152]. The expression of MCT4 in the stroma of triple-negative breast tumors is associated with a poor prognosis [153]. However, the opposite has also been described: lactate is taken up from the interstitium by myofibroblasts through the anion exchange protein 2 (AE2) transporter [154], making the interactions of myofibroblasts with interstitial lactate more complex than initially thought.

Due to the lavish expenditure of amino acids that tumor cells make in order to proliferate, as already indicated, autophagy is activated in CAFs. Protein catabolism in the CAFs releases amino acids into the extracellular space, thereby maintaining the availability of amino acids in the interstitium. In this way, these amino acids are captured by tumor cells [50,51,155]. For example, alanine serves as an anaplerotic substrate to maintain TCA in tumor cells [51].

Autophagy activation in CAFs occurs after detecting a lack of nutrients such as glucose and amino acids. The most critical activator of CAFs is the oxidative stress produced by reactive oxygen species (ROS) derived from the relative hypoxia of the tissue, arising from its hypovascularization and from the decrease in glutathione synthesis derived from the deficit of its amino acid components (glutamine, cysteine, and glycine). This leads to a drop in the levels of P62, which is a negative regulator of autophagy [156]. In this way, activating transcription factor 4 (ATF4) is stabilized, and the secretion of NEAAs to the interstitium is favored [155]. Interestingly, low P62 levels have been observed in the stroma of a variety of tumors [156].

Finally, it should be noted that the lack of oxygen also contributes to the functional changes in fibroblasts that are ultimately directed to allow tumor growth. Specifically, hypoxia induces (i) the HIF1-α that activates genes related to the process of fibrosis in CAFs [157,158]; (ii) the expression of TGF-β [159,160]; and (iii) autophagy [161]. Assuming that the process of oxygen deprivation and the lack of glucose persist, then there is a deleterious effect on CAFs, and a decrease in αSMA-positive stromal cells [162] by delaying the survival of CAFs, which contributes to the tumor necrosis so frequently observed in many tumors.

#### 2.1.2. Metabolic Changes in Adipocytes

The adipocytes surrounding cancer cells undergo functional changes that favor tumor growth [163]. Cancer-associated adipocytes are characterized by increased brown/beige fat markers and catabolic activity. Many of the catabolites generated are released into the interstitium and used by cancer cells to facilitate tumor growth and progression. Lactate, pyruvate, ketone bodies, and free fatty acids are among the metabolites released [164]. Specifically, ketone bodies are transformed into ATP in the mitochondria more easily than other substrates, which favors tumor cell proliferation under conditions of low blood perfusion [165]. This also explains how the coexistence of adipocytes and tumor cells enhances ketogenesis in adipocytes and ketolytic activity in cancer cells [164]. Additionally, the high level of expression of genes related to ketogenesis has been related to a worse prognosis in breast cancer patients [166]. However, the involvement of ketone bodies in fueling TCA is somewhat controversial since it has mainly been described in vitro and in some xenografts [167,168]. Paradoxically, the ketogenic diet has been proposed as a cancer treatment [169].

Cancer-associated adipocytes have also been found to release β-hydroxybutyrate, which induces the expression of protumoral genes in breast cancer cells in vitro [170]. Adipocytes may also favor more advanced stages of cancer, such as spreading. Indeed, elevated levels of phosphatidylinositol transfer protein, cytoplasmic 1 (PITPNC1), in adipocytes are associated with a high prevalence of omental metastasis and with poor prognosis in gastric cancer [171].

In the context of breast cancer, changes in the adipocytes surrounding tumor cells have been observed. They exhibit reduced expression of adipocyte markers and a lower lipid content. Additionally, adipocytes become activated and overexpress inflammatory proteases and cytokines [163,172]. When in contact with adipocytes in vitro, triple-negative breast cancer cells boost the expression of genes encoding proteins with greater proinflammatory activity and migration capacity [173]. Adipocytes also play a role in resistance to chemotherapy with doxorubicin and other drugs, contributing to the appearance of a multi-resistant phenotype [174,175]. This effect has been associated with the expression in adipocytes of the transport-associated major vault protein (MVP) that is associated with significantly greater elimination of drugs from tumor cells through vesicles, fundamentally at the invasion front [175].

#### 2.1.3. Metabolic Changes in Endothelial Cells

Endothelial cells are essential in tumor angiogenesis, and it is through the new vessels that tumor spread begins. Indeed, although tumors can use pre-existing vessels for their spread (vascular cooption), the abnormal tumor vessels generated can be gateways for the spread of tumor cells [176,177]. Endothelial cells in blood vessels have a high requirement for glucose, especially during VEGF-promoted angiogenesis, compared with quiescent endothelial cells in already formed vessels. Thus, the shortage of glucose in the interstitium can contribute to defective tumor angiogenesis and the presence of hypovascularized areas in tumors [178]. Tumor neovascularization, therefore, depends on the availability of glucose [179].

Paradoxically, interstitial lactic acidosis also stimulates angiogenesis. Lactate induces NFκB and HIF1α [180,181]. It has been proposed that glucose availability initiates neoangiogenesis, but the presence of lactate allows the subsequent maturation of the blood vessels. Lactic acidosis prompts a stress response in the endoplasmic reticulum of endothelial cells that favors their survival [182,183].

As in other cell types, endothelial cells also depend on the availability of NEAAs. Like glucose shortage, the deficiency of NEAAs in the interstitium contributes to the angiogenesis defect present in tumors. Specifically, glutamine is essential in endothelial cells for (i) generating new vessels [184,185], (ii) restoring TCA in an anaplerotic reaction, and (iii) asparagine synthesis. Glutamine and the other defective NEAAs contribute to insufficient angiogenesis. For example, glycine is required in order for VEGF to exert its angiogenic function [186], and serine is crucial for mitochondrial function in endothelial cells [187].

Molecular crosstalk has been described between breast cancer tumors and endothelial cells, which help regulate angiogenesis and tumor dissemination [188]. It has also been suggested that, independently of their angiogenic capacity, endothelial cells can help breast cancer cells to survive under conditions of low nutrients in vitro and to maintain their stem cell properties (stemness) [189]. Therefore, it is likely that endothelial cells help create the tumor niche where the environmental conditions exist that are necessary to maintain stemness and initiate the tumor. Another action exerted by endothelial cells, independent of new vessel generation, promotes breast cancer metastasis. Thus, in TNBC cells treated with TGF-β, the epithelial-to-mesenchymal transition (EMT) phenotype and the production and secretion of plasminogen activator inhibitor 1 (PAI-1) are induced. PAI-1 induces the production of CCL5 in endothelial cells, creating a positive feedback mechanism that causes the dissemination and production of more PAI-1 breast cancer cells [190].

### 2.2. Metabolic Changes in Stromal Cells of Leukocyte Origin

The tumor stroma comprises several leukocyte cell subpopulations, the best studied of which are T lymphocytes and macrophages. However, other cells of myeloid origin can also infiltrate tumors. The existence of tumor-associated neutrophils (TANs), like macrophages, could have an antitumor or protumoral function [191]. Tumor infiltration by TANs is associated with poor prognosis in several tumor subtypes [192] and has been described in the evolution from papilloma to carcinomas in a chemical carcinogenesis model [193]. However, its role in tumor prognosis, including breast cancer, is not well defined [194]. Dendritic cells of myeloid origin are antigen-presenting cells and, they make up part of the phagocytic mononuclear system. They are known to infiltrate tumors. As such, they are required for the immune response to cancer [195]. Immature and mature forms have both been described in tumor infiltration. Infiltration of mature dendritic cells is associated with increased aggressiveness of breast cancer [196].

In this review, we will mainly focus on T lymphocytes and macrophages.

#### 2.2.1. Metabolic Changes in T Lymphocytes

##### Different Lymphocyte Subpopulations and Their Role in Tumor Evolution

Before reviewing the metabolic changes observed in T-cells, it is worth recalling their role in tumor pathogenesis, especially in the context of breast cancer. In general, within tumor-infiltrating lymphocytes (TILs), T lymphocytes with cytotoxic or proinflammatory activity are associated with the good evolution of breast and other cancers, while those with anti-inflammatory and suppressive activity favor tumor growth and poor evolution. Thus, in breast cancer, cytotoxic T lymphocytes (CD8+) are the most abundant. Their high degree of infiltration is associated with a better prognosis and complete response to neoadjuvant chemotherapy [197,198,199,200]. Conversely, a low level of infiltration is associated with a higher risk of metastasis [201]. Certainly, CD8+ T-cells can directly kill tumor cells by releasing cytolytic enzymes and inducing apoptosis, making them essential to the antitumor immune response [202]. CD8 cytotoxic cells use two strategies to kill cancer cells: death ligands (such as TNFα, FAS ligand, and TRAIL) and granule exocytosis [203,204]. The latter CD8 cells release a pore-forming protein (perforin) that delivers serine-proteases, the granzymes, into the cytosol of the target cells responsible for eliminating them. CD8 T-cell levels can also be low in tumors, as in the case of squamous cell carcinoma [205], which is favored because the TGF-β present inhibits its infiltration of the tumor and its function by favoring the expression of T-cell exhaustion markers such as PD1, CTLA4, and Tim-3 [206,207].

The extensive infiltration of the tumor by T-helper 1 CD4+ (TH1) lymphocytes, which produce INFɤ, and other molecules with pro-inflammatory activity, is associated with a good prognosis in breast cancer [208]. In contrast, the tumor infiltration by lymphocytes with TH2, with anti-inflammatory, suppressive, and pro-regenerative activity, is associated with the poor evolution of breast cancer due to the induction of metastases [209]. Therefore, TH17 lymphocytes, which are proinflammatory and involved in the antitumor response, are associated with a good prognosis [210]. In comparison, FOXP3+ Treg lymphocytes that participate in immunosuppression and tissue tolerance are associated with a worse prognosis in breast cancer [211,212,213]. Treg lymphocytes are higher in HER2-positive breast cancer than in HER2-negative tumors [214]. Natural killer (NK) cells are members of the family of innate lymphoid cells. They have cytotoxic and proinflammatory activity in tumors, whereby they can inhibit local and distant tumor growth and eliminate incipient tumor cells (immunosurveillance) [215]. Therefore, its abundance in the tumor stroma is associated with the excellent evolution of breast cancer and is positively correlated with the neoadjuvant chemotherapy response [216].

##### Metabolic Influences on Lymphocyte Functions in Tumors

Due to the high level of glucose consumption by tumor cells, sugar is also scarce in the interstitium for T lymphocytes. Effector T-cells are very sensitive to glucose deficiency [10,217,218]. Thus, the synthesis of INFɤ in CD4+ naïve lymphocytes is disrupted without glucose, producing a defect in their differentiation to TH1 cells [217]. The differentiation and growth of regulatory TH2 cells are, therefore, favored [219,220,221]. Ultimately, the suppressive function of T lymphocytes in the tumor predominates over their proinflammatory and effector antitumor response. The scarcity of glucose in the interstitium is critical to this process. The PD1/PDL1 interaction exerts its suppressive function on T lymphocytes by this mechanism. The PD1 receptor on activated T lymphocytes suppresses glucose uptake [222]. Conversely, blocking PD1/PDL1 with antibodies increases GLUT1 levels and glucose uptake by T lymphocytes and bestows an antitumor phenotype [217].

Interstitial lactate acidosis also favors this defect in differentiation to proinflammatory TH1 cells [10]. Extracellular lactate depresses the function of effector T-cells [223,224]. Extracellular acidity also ends up being translated into intracellular acidity and, due to the action of the 2-hydroxy-glutarate produced by LDH-A, inhibits the translocation of NFAT to the nucleus [225]. In turn, this reduces the translation and synthesis of INFɤ and other specific genes of the proinflammatory T lymphocytes [223,226].

In the context of breast cancer, interstitial lactic acidosis inhibits the release of lactate from T lymphocytes by inhibiting the MCT1 transporter. The accumulation of lactate within the TILs also inhibits glycolytic activity, giving rise to a lower energy and biosynthesis capacity, leading to less proliferation, cytolytic capacity, and cytokine production [224,227,228]. The functional deficiency due to lactate acidosis also affects the NK cells [223]. Moreover, other innate lymphoid cells (ILCs) have been described in addition to NK cells, such as ILC1, ILC2, ILC3, and lymphoid tissue-inducer (Lti) cells. Like NK cells, all of them are similar to T-cells in their appearance and function. However, they lack the T-cell receptor (TCR) [229,230]. The role of these subpopulations in tumor development and rejection is under study, and they could prove to be new targets for immunotherapy [231]. Indeed, it has recently been reported that lactic acidosis of the interstitium depresses the function of ILC2 cells. In addition, stimulating ILC2 cells with Il-33 facilitates the rejection of melanoma cells [232].

The proliferation of T lymphocytes is sensitive not only to the lack of glucose in the interstitium for their proliferation but also to the lack of amino acids [233]. In addition, in the absence of glutamine, the proliferation of T-cells decreases and, as with the other stimuli of the interstitium (the lack of glucose and the increase in lactate), a change in cell function is induced from effector to suppressor regulatory T-cells [234]. Competition for glutamine in the interstitium between tumor cells and TILs dampens their proinflammatory capacity and leads to a predominantly suppressive and pro-regenerative change in T-cell functional activity. This change also affects T-cell subpopulations such as TH17, which change to exhibit Treg activity [234]. Glutaminase (GLS) transforms captured glutamine into glutamic acid. A high level of GLS expression in tumor cells of TNBC is associated with significant uptake of glutamine from the interstitium and decreases in the glutamine available for TILs and in T-cell infiltration. Conversely, the strong expression of GLS in TILs themselves is associated with significant infiltration of these cells in the tumor [235].

In addition to the lack of glutamine, the shortage of other amino acids is critical. The lack of tryptophan and its transformation by tumor cells and CAFs into kynurenine, which induces the differentiation of T effector cells to a regulatory phenotype, contributes to this [236,237]. Arginine deficiency also limits the proliferation of T lymphocytes [238,239]. The uptake by T lymphocytes of other amino acids, such as serine, is necessary for the de novo synthesis of nucleotides, and its deficit also disrupts the proliferation of T lymphocytes [240].

The hypoxic state of the interstitium also contributes to the immunosuppression of T lymphocytes. Hypoxia stabilizes HIF1-α and enhances the cytotoxic function of T-cells [241], but when it persists, the activation of T lymphocytes is limited [242], the effect being enhanced by glucose deficit [243].

#### 2.2.2. Metabolic Changes in Macrophages

It is essential to note that, with respect to macrophages, immune suppressor cells in the tumor are associated with a poor prognosis [244,245,246]. From a functional point of view, several types of macrophages can be identified in the tumor. We describe these below in the context of breast cancer.

##### Types of Macrophage in the Tumors

From a functional point of view, there are two spectra of tumor-associated macrophage (TAM) in tumors: (i) M2-like TAMs, which have anti-inflammatory and regenerative activities that arise from cytokines derived from TH2 lymphocytes such as TGF-β, IL4, IL10, and IL13; and (ii) M1-like TAMs, which have inflammatory and antitumor activity and are produced in response to the INFɤ produced by TH1 lymphocytes. Other authors consider that the concept of TAMs encompasses only those whose M2 activity promotes tumor development while the M1 participates in tumor rejection [247]. According to the so-called immunoediting theory, tumor progression consists of a series of phases, beginning with the elimination of antigenically different tumor cells, reaching an equilibrium, and finally, effecting an escape [248]. M1 macrophages participate primarily in the elimination stage of antigenically different tumor cells, but progress through all three stages is made possible by changes in the M1/M2 ratio [248]. A high degree of infiltration of anti-inflammatory TAM M2 is associated with poor prognosis in cancers, including breast cancer [249,250]. Specifically, tumor infiltration by M2 CD163+ macrophages in breast cancer is associated with larger tumors, a higher recurrence rate, a higher histopathological grade, and ER-negative tumors that are more aggressive than ER-positive ones [251,252,253,254].

##### Metabolic Influences on Macrophage Functions in Tumors

The metabolic conditions of the tumor favor the functional transformation of M1 into M2 macrophages. The acidosis of the interstitium itself suppresses several aspects of the proinflammatory function of macrophages, such as migration, cytokine production, and antigen presentation. In the end, the proinflammatory function is depressed, and differentiation to an M2 state with immunosuppressive activity is favored, facilitating the proliferation of the tumor parenchyma [238,255,256].

Lactate induces TAM polarization to an M2 phenotype through their G-protein-coupled receptor, GPR132. A high level of GPR132 expression in breast tumors is positively correlated with TAM infiltration, metastasis, and, thereby, poor outcome [257]. In breast cancer, M2 TAMs can directly influence the behavior of tumor cells, whereby TAMs, under the influence of lactate, express and release CCL5 protein via NOTCH, which binds to its receptor in breast cancer cells and induces glycolysis and their epithelial–mesenchymal transition [258].

Increased ROS production by macrophages induces their transformation into the M2 phenotype. Indeed, their proinflammatory activity ensures that macrophages produce a large quantity of ROS, which are buffered by the NADPH generated by the PPP [259,260] and by intracellular glutathione. In the absence of amino acids such as serine and cysteine, macrophages cannot regenerate sufficient NADPH and glutathione, so the ROS are significantly augmented at the intracellular level, which causes a loss of the antitumor M1 phenotype [261]. Arginase 1 produced by anti-inflammatory M2 macrophages contributes to increased arginine deficiency, limiting the proliferation of T lymphocytes [238,239]. Arginine is handled differently in this functional change of macrophages. The M1 cells, with their proinflammatory function, produce nitric oxide (NO) and citrulline from arginine through the action of inducible nitric oxide synthase (iNOS) [262]. In contrast, the M2 cells produce ornithine and urea through the action of arginase-1. These actions are consistent with the decrease of citrulline in TNBC, the low level of expression of iNOS, and the production of NO [263].

Hypoxia also contributes to macrophage immunosuppression. It induces HIF1-α, a phenotype that favors the appearance of the proinflammatory M1 phenotype in macrophages [264]. However, the deficiency of glucose and oxygen causes the macrophages to generate a suppressive and pro-regenerative M2 phenotype, which expresses arginase 1 [180,265]. These events account for the abundance of immunosuppressive macrophages in hypoxic tumor areas [266]. TAMs isolated from hypoxic areas of mouse breast tumors have lower glycolytic activity, with lower glucose utilization being associated with greater glycolytic activity in endothelial cells and with angiogenesis [267].

**Figure 2:**
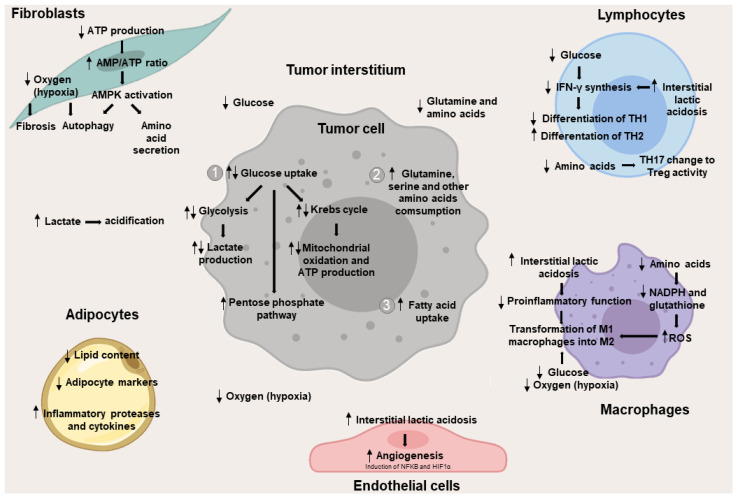
Resume of the metabolic changes taking place in tumor cells and interstitial cell subpopulations. Lactate is released into the interstitium, contributing to its acidification. The acidity and hypoxia of the interstitium and the relative deficit of glucose and amino acids induce functional changes in the various cell subpopulations of the interstitium, including myofibroblasts, endothelial cells, T lymphocytes, and macrophages, among others. All these changes mainly promote tumor growth. Upward pointing arrows indicate increased levels or activity of that molecule or pathway. The downward pointing arrows indicate the opposite. Created using BioRender.

Some of the main metabolic changes described so far are summarized in Figure 2.

## 3. Towards a Pathophysiological and Functional Integration of Metabolic Changes in the Parenchyma and Tumor Stroma: The Reverse Warburg Effect or Metabolic Coupling Model

### 3.1. Criticisms of the Universality of the Warburg Effect

The energy of normal cells comes mainly from glucose, which is catabolized and transformed to pyruvate in the glycolysis process in the cytosol. After this, pyruvate enters the Krebs cycle or the TCA cycle, which occurs in the mitochondria. Here, through the respiratory chain, energy is generated in the form of ATP with oxygen expenditure, and CO_2_ and water are formed as the final products of the reaction. The energy production mechanism through TCA with oxygen expenditure is very efficient. However, in the absence of oxygen, pyruvate does not enter the TCA but is transformed into lactate, and the production of ATP is 19 times lower.

Therefore, the observation that the production of low-yield energy predominates in cancer because the pyruvate acetyl groups do not enter the TCA is surprising. This happens under physiological conditions in the absence of oxygen, the pyruvate subsequently being transformed into lactate. However, this occurs in tumors, even in the presence of oxygen. This reprogramming of tumor metabolism is considered one of the fundamental characteristics of cancer [268]. As indicated in the first part of the review, Otto Warburg was the first to observe the predominance of aerobic glycolysis at the expense of oxidative phosphorylation in tumors [1], for which reason the pattern has come to be known as the Warburg effect.

An initial hypothesis to explain the Warburg effect proposed that the tumor cells contained defective mitochondria, obliging them to use aerobic glycolysis [269] and that the repression of oxidative phosphorylation (OXPHOS) was obligatory for the proliferation of tumor cells. However, this proved not to be the case: tumor cells do not have defective mitochondrial function, and OXPHOS repression is not essential for tumor cell proliferation [270]. Moreover, OXPHOS activity is elevated in cells of various tumor types, including various forms of leukemia and lymphoma [271], pancreatic cancer [272], and melanoma [273].

### 3.2. An Alternative Model: The Reverse Warburg Effect or Metabolic Coupling Model

**Figure 3:**
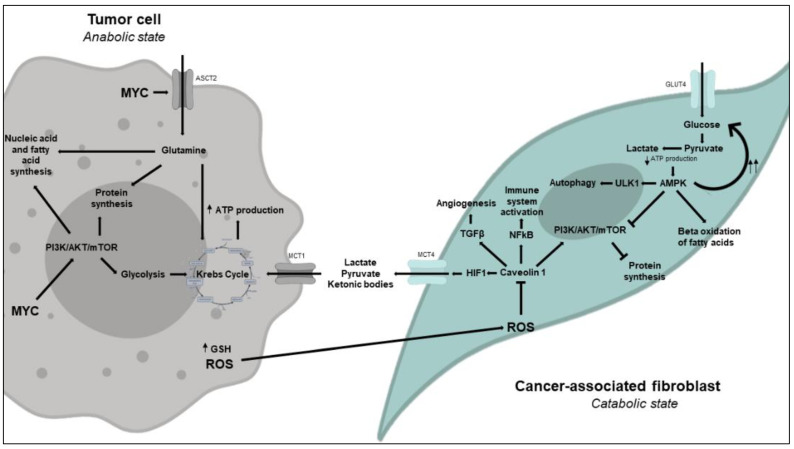
Schematic representation of the coupling model or reverse Warburg effect. The coupling model proposes the integration of the various metabolic changes observed in cancer in a more functional manner. This model is based on the premise that most human tumors, such as those of the breast, stomach, and pancreas, are comprised of stroma. The Warburg effect preferentially occurs in the predominant stromal cell type, i.e., the CAFs, where it manifests as an increase in aerobic glycolysis and a hypofunctional tricarboxylic acid (TCA) cycle. This leads to a significant release of lactate and ketone bodies into the interstitium and their capture by tumor cells. Once inside the cells, these molecules feed and enhance TCA activity. Likewise, the activation of autophagy in CAFs releases a large quantity of amino acids into the interstitium, which is captured by the cells of the tumoral parenchyma for use in the anabolic synthesis of protein. Catabolic reactions, therefore, predominate in the stromal CAFs, favoring the preponderance of anabolism in the tumor cells. This is known as the reverse Warburg effect or the coupling model. Upward pointing arrows indicate increased levels or activity of that molecule or pathway. The downward pointing arrows indicate the opposite. Created using BioRender.

Initially, the Warburg effect was attributed to the entire tumor and specifically to cancer cells. However, many tumors have an important stromal component, and a more detailed study proposed that in many cases, the Warburg effect does not occur in the tumor cells but, instead, in the stroma. According to this model, which has been called the reverse Warburg effect, a symbiosis is established between stromal and tumor cells that allows both cell populations to grow, for which reason it is also referred to as the coupling model, in which the metabolic behavior differs between CAFs and tumor cells [274]. Below, we will revise again the metabolic changes in myofibroblasts and tumor cells based on the coupling model, then discuss some aspects of this model in breast cancer. The interactions between tumors cells and CAFs in the coupling model are summarized in Figure 3.

#### 3.2.1. Metabolic Changes in CAFs

##### An Excess of ROS Produced and Released by Tumor Cells Induces Metabolic Reprogramming in CAFs to Enable Aerobic Glycolysis

CAFs can be metabolically reprogrammed by an excess of free radicals emerging from tumor cells. In particular, this involves hydrogen peroxide production, which is also essential for wound healing. The excess ROS of tumor cells arises from their metabolism and can be induced by activating oncogenes and the loss of suppressor genes [133,275,276]. Antioxidant pathways and the production of reduced glutathione are activated to prevent damage to tumor cells [277,278]. This response is similar to that occurring in tissues under fasting conditions and without oxygen [279]. The metabolic reprogramming of the Warburg effect in CAFs assumes that the lack of entry of pyruvate into the Krebs cycle causes a defect in the respiratory chain and subsequent oxidative phosphorylation and, consequently, the production of less ATP in these cells.

##### Low ATP Levels in CAFs Activate AMPK and Have Fasting-Like Metabolic Consequences in Response to an Increased Catabolic State

As already indicated in the previous section, the change in AMP/ATP ratio, with a relative increase in the intracellular concentration of AMP, leads to the activation of AMP-kinase (AMPK), which sets in motion an activation response of pathways that partly coincides with those occurring in the cell under conditions of glucose deficiency (fasting). Thus, (i) glucose uptake is favored, with increased synthesis of the GLUT4 transporter; (ii) the activity of the glycolytic pathway is enhanced by phosphorylation of the enzyme phosphoglucokinase 2; (iii) β-oxidation of fatty acids is also activated by AMPK after it has inhibited β-acetyl-CoA carboxylase; (iv) AMPK also activates autophagy pathways to facilitate the recycling of cellular materials by phosphorylating Unc-51-like kinase 1 (ULK1) [280]; and (v) protein synthesis is inhibited in CAFs by inhibition of mTOR by AMPK.

##### Inhibition of Caveolin 1 by ROS Promotes Fibroblast Differentiation into Myofibroblasts (CAFs)

The differentiation of fibroblasts to myofibroblasts (CAFs in tumors) is carried out by oxygen free radicals, otherwise known as reactive oxygen species (ROS), mainly hydrogen peroxide produced by the tumor cells. ROS inhibit caveolin 1. This is an essential step in myofibroblast differentiation [281]. Caveolins are proteins that are essential for maintaining the structure of caveolae, which are invaginations of the plasma membrane containing lipid rafts characterized by the abundance of sphingolipids, cholesterol, and signaling proteins. Within the family of caveolins, caveolin 1 (CAV1) inhibits, through its scaffolding domain, many signaling proteins such as G proteins, SRC family kinases, RAS family proteins, and endothelial nitric oxide synthase (eNOS), among others. The proliferation of the tumor parenchymal cells releases a huge quantity of free radicals, from which the tumor cells defend themselves with a high rate of synthesis of NADPH via the PPP and glutathione synthesis. Free radicals diffuse from cancer cells to the interstitium and induce the formation in the fibroblasts of phagolysosomes, which degrade CAV1. The loss of CAV1 stops the phagolysosomes inhibiting the signaling pathways that contribute to (i) the differentiation of fibroblasts into myofibroblasts by TGF-β; (ii) the generation of a catabolic metabolism with elevated glycolysis and the release of lactate into the interstitium; (iii) the various aforementioned functional changes, which appear in myofibroblasts (protein production by autophagy, generation of the stromal matrix, and others). The loss of CAV1 in the stroma is associated with poor prognosis in breast cancer and other tumors such as melanoma and those of the pancreas, esophagus, prostate, and stomach [281].

CAV1 loss increases the signaling of fundamental pathways of stromal function, such as PI3K/AKT/mTOR, TGF-β, NFκβ, and HIF1α. Indeed, TGF-β is essential to angiogenesis, and NFκβ produces a multitude of cytokines that facilitate the activation of the immune system at the local level. HIF1α performs many functions, in particular, increasing the synthesis of the MCT4 transporter that allows the metabolites such as pyruvate, lactate, and ketone bodies to exit the CAFs. These molecules are captured from the interstitium by tumor cells, thanks to the MCT1 transporter, giving rise to anaplerotic reactions that enhance the TCA cycle in them [282,283]. Therefore, the defect in the Krebs cycle of CAFs enhances that of tumor cells [274].

The phenomenon of coupling between CAFs and cancer cells, for example, in breast tumors, has also been noted at the lipid metabolism level. CAFs synthesize many fatty acids, thanks to the overexpression of the fatty acid synthetase (FAS), and are released into the interstitium. From there, tumor cells take up fatty acids, which, unlike CAFs, have low levels of FAS and fatty acid synthesis. However, there is an increase in fatty acid uptake because they express high levels of the FATP1 transporter [284].

#### 3.2.2. Metabolic Changes in Tumor Cells

As previously indicated, while the anaerobic glycolysis state predominates in stromal cells, with little ATP production and an increased catabolic state, an anabolic state of protein synthesis predominates in tumor cells, featuring a high level of ATP synthesis due to increased oxidative phosphorylation. The latter increases due to the greater activity of the tricarboxylic acid cycle, which results from the uptake of pyruvate, lactate, and ketone bodies from the stroma, which, in turn, is derived from the stromal Warburg effect.

The Myc pathway favors tumor proliferation. Indeed, a key element of the increased proliferation of stromal cells comes from the direct or indirect increase in the activity of the Myc pathway, which, for example, is activated in more than half of breast tumors. Myc activates the PI3K/AKT/mTOR pathway in the tumor cell, leading to an increase in protein synthesis and glycolysis with final oxidative phosphorylation. Along with protein synthesis, Myc generates ribosomes, mitochondria, and nucleotides to synthesize nucleic acids and fatty acids, all of which are cell membrane constituents [285].

##### Glutamine Metabolism Is Essential to Tumor Cell Proliferation

Glutamine metabolism also plays an essential role in tumor cell proliferation. Glutamine enters cells through the alanine-serine-cysteine transporter 2 (ASCT2), whose synthesis is induced by Myc. Once inside the cell, glutamine has several possible fates: (i) to participate in nucleotide synthesis; (ii) to participate in protein synthesis; (iii) to generate or restore glutathione levels; (iv) to enter the TCA cycle. In the latter case, it participates in synthesizing the oncometabolite 2-hydroxy-glutarate [286].

The role of glutamine in the Krebs cycle is essential for tumor cells. Glutamine is transformed into glutamic acid by the action of glutaminase (GLS), whose synthesis is induced by MYC through the action of many miRNAs [287,288]. Glutamic acid is transformed into alpha-ketoglutaric acid (α-KG), which is a component of the TCA cycle. α-ketoglutarate is transformed by mitochondrial IDH1 (isocitrate-dehydrogenase) or by cytoplasmic IDH2 into 2-hydroxy-glutarate (2HG). This molecule is considered an oncometabolite.

Several tricarboxylic acids involved in the Krebs cycle are also considered oncometabolites, such as succinate, fumarate, and 2HG, which is derived from alpha-ketoglutarate. Their involvement as oncometabolites was shown in diseases in which these products accumulate innate errors of metabolites [289]. Another proof of the oncogenicity of these metabolites is that the enzymes generating them, isocitrate-dehydrogenases 1 and 2 (IDH 1 and 2), appear mutated, with a gain of function in some tumor processes, such as those of gliomas and leukemias [290,291]. They all inhibit the alpha-KG-dioxygenase pathways, resulting in epigenetic changes that give rise to a stemness phenotype.

In summary, it appears that the purpose of blocking the entry of pyruvate into the Krebs cycle of the stromal cell is partly to increase the amount of pyruvate available for the Krebs cycle of the tumor cell. Therefore, the Warburg effect of the stroma, in which catabolism and defective ATP synthesis predominate, is accompanied by an enhancement of the Krebs cycle in the tumor cells, preferentially with anabolic activity and an increase in ATP synthesis. This metabolic symbiosis is known as the stroma–tumor cell coupling pattern.

### 3.3. The Coupling Model in the Context of Breast Cancer

As the coupling model suggests, in breast cancer, the metabolic reprogramming in CAFs with low caveolin levels is associated with increased catabolism and the production of lactate, glucose, and ketones that are released into the interstitium, where they serve as nutrients for breast cancer tumor cells. The coupling model in breast cancer has been demonstrated by studies of co-cultures of CAFs and tumor cells. Thus, independent gene expression studies showed that tumor cells overexpressed TCA and mitosis genes, while CAFs overexpressed genes encoding glucose-binding proteins and aerobic glycolysis enzymes [292]. Furthermore, CAFs with high glycolytic activity promote tumorigenesis in vitro [293] and in vivo [294] and induce resistance to antiestrogenic treatment in ER-positive tumor cells [295].

Another proof of the existence of a metabolic coupling in breast cancer derives from tracing the destiny of metabolites between tumor cells and CAFs in co-cultures, such as MDA MB231 cells co-cultured with CAFs. MDA MB231 releases lactate, which is taken up by CAFs [296,297], transforming it into metabolites such as pyruvate, which are exported to the environment and captured by tumor cells to obtain energy [297].

Therefore, it has been proposed that breast cancer with low levels of caveolin can be treated with inhibitors of glucose and lactate transporters. Inhibiting the glycolytic pathway in breast cancer xenografts has been shown to reduce tumor growth significantly [192].

### 3.4. Criticisms of the Universality of the Reverse Warburg Effect

Although the coupling pattern occurs in several tumor types [150,298], no single model explains the metabolic interactions between stromal cells and tumor parenchymal cells. For example, in breast cancer, the behavior of CAFs varies with the tumor subtype of origin. Thus, CAFs isolated from luminal tumors have the paradoxical effect of inhibiting glucose uptake in the MCF7 luminal cell line while having no effect on basal cell lines. Conversely, CAFs extracted from basal tumors favor glucose uptake in tumor lines of basal and luminal origins [19].

It is very probable that no single model of metabolic interactions between the various cellular components of tumors will be able to explain all the observed patterns.

## 4. Conclusions

Metabolic studies in cancer reveal the extraordinary plasticity of tumor cells’ ability to acquire nutrients that allow them to proliferate and survive, usually in adverse circumstances resulting from a perfusion deficit [299]. To achieve this, cancer cells induce metabolic and functional changes in the surrounding stroma cells that favor tumor growth and are associated with tumor progression and prognosis. Indeed, stromal cell subpopulations such as CAFs, TAM, or TILs are associated with prognosis in breast cancer and other tumors. The metabolic changes observed in cancer show alterations in enzymes, metabolic mediators, and end products and the flux of metabolites. Among them is the potentiation of glycolysis, TCA activity, and glutaminolysis and an increase in lipid synthesis pathways; there is also a depletion of glucose, oxygen, and amino acids in the interstitium, alongside lactate accumulation.

These metabolic changes are not homogeneous in tumors; there are differences between types of cancer originating in different tissues and within the same tissue, such as breast cancer, where metabolic alterations have different nuances depending on the intrinsic subtype of breast cancer and the degree of differentiation [300]. Additionally, in vivo studies reveal the metabolic heterogeneity of cancer throughout tumor progression [11,301], whereby they are more dependent on OXPHOS metabolism in more advanced stages [91]. Indeed, OXPHOS inhibitors have been proposed for use in treating cancer and are undergoing clinical trials at the time of writing [302]. Moreover, given the tumors’ addiction to glutamine, the inhibition of glutaminase with glutamine analogs has been proposed as a means of treating cancer. Indeed, glutamine analogs are already undergoing clinical trials [303].

Given the importance of metabolic changes in the progression and prognosis of cancer, a better understanding of them may lead to new findings and vulnerabilities that allow better treatments of the disease. The metabolic fluxes identified through in vitro studies have provided essential information; however, they have limitations. The metabolic behavior of tumors differs in vivo and in vitro [304], so more in vivo studies are needed to understand better how metabolic changes contribute to establishing functional changes in stromal cell subpopulations and the bidirectional metabolic crosstalk between tumor and stromal cells. The systemic administration of metabolic tracers labeled with carbon-13 or other radioisotopes may, in the future, determine which patients may benefit from the inhibition of specific metabolic pathways [11]. For example, PET tracers can detect in vivo OXPHOS and the glutamine dependence of tumors, making them susceptible to treatment with inhibitors of these metabolic activities [305,306]. In this regard, it is expected that advances in stable isotopic tracers, imaging-based assays, and new mass spectrometry tools will confirm and expand metabolic discoveries previously made in vitro and identify new vulnerabilities that will lead to new therapeutic targets for more individualized cancer treatments.
